# Venous thromboembolism after adult thymus or thymic tumor resection: A single‐center experience

**DOI:** 10.1111/1759-7714.13543

**Published:** 2020-06-18

**Authors:** Xingguo Yang, Lei Yu, Tao Yu, Fei Li, Yunfeng Zhang, Zhen Yu, Baoxun Zhang, Ji Ke, Hui Li

**Affiliations:** ^1^ Department of Thoracic Surgery, Beijing Tongren Hospital Capital Medical University Beijing China; ^2^ Department of Thoracic Surgery Beijing Chaoyang Hospital, Capital Medical University Beijing China

**Keywords:** Deep vein thrombosis, thoracic surgery, thymectomy, venous thromboembolism

## Abstract

**Background:**

Venous thromboembolism (VTE) is a common postoperative complication. Previous studies have shown that the VTE incidence after major thoracic surgery is high. However, there have been no exclusive data after thymectomy thus far. To investigate the incidence of postoperative VTE, we conducted a single‐center, prospective cohort study.

**Methods:**

Patients who underwent thymectomy between December 2017 and January 2020 were enrolled. None of the patients received any prophylaxis perioperatively. Subjects were risk stratified into groups of low risk (0–4), moderate risk (5–8), and high risk (≥9). Occurrence of VTE events, including deep vein thrombosis (DVT) and pulmonary embolism (PE), were identified by imaging.

**Results:**

There were 192 patients who underwent thymectomy enrolled into the study. The overall VTE incidence was 8.9%. All the patients were diagnosed with DVT, and none were diagnosed with PE. The VTE incidence was 4.6% in patients with benign thymic diseases and 14.5% with malignant diseases. The VTE incidence was 4.7% in patients undergoing thoracoscopic surgery and 22.7% undergoing median sternotomy. The VTE incidence increased with Caprini score. Scores in the low, moderate, and high risk groups were associated with a VTE incidence of 0%, 10.3% and 37.5%, respectively. In patients with thymic malignancy, the VTE incidence in the moderate and high risk groups were 8.8% and 31.8%, respectively.

**Conclusions:**

VTE occurred frequently in patients after thymectomy without VTE prophylaxis. The median sternotomy procedure and malignant tumor may be the major risk factors for the development of VTE. Aggressive VTE screening/treatment protocols should be implemented in patents after thymectomy.

## Graphical abstract

In this paper, we evaluated the incidence of VTE after adult thymus or thymic tumour resection. We found that VTE occurred frequently in patients after thymectomy without VTE prophylaxis, and the median sternotomy procedure and malignant tumor may be the major risk factors for the development of VTE.

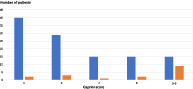

## Introduction

Venous thromboembolism (VTE), including pulmonary embolism (PE) and deep vein thrombosis (DVT), is one of the most serious and preventable complications after major surgical procedures^.^ ^1^ DVT and PE are important causes of unexpected death in hospitals. Without thromboprophylaxis, the incidence of objectively confirmed DVT is 10%–40% in general medical and surgical patients. However, the clinical burden of postoperative VTE after thoracic surgery is likely underestimated because most patients are asymptomatic or misdiagnosed^.^ ^2^ Thrombotic complications are the most common cause of 30‐day postoperative mortality^3^, ^4^, ^5^and contribute to prolonged lengths of hospital stay and increased hospital costs.^6^, ^7^

In a recent single‐center prospective cohort study, Li *et al*. found that the overall incidence of VTE was 13.9% after major thoracic surgery without perioperative VTE prophylaxis and the incidence of VTE after thoracic cancer surgeries was as high as 17.5%.^8^ Reported risk factors of thrombosis related to thoracic cancers included age, sex, BMI >25 (kg/m^2^), swollen legs, the presence of venous varicosities, a history of VTE, a prolonged duration of surgery, receipt of chemotherapy therapy, and immobility.^9^, ^10^, ^11^ A sufficient amount of research describes the incidence of VTE following elective thoracic surgery, and it is also well documented that lung and esophageal cancer surgery are specific risk factors for VTE compared to other elective thoracic surgery procedures.^12^, ^13^ However, no studies have described the incidence of VTE after thymectomy.^10^

The Caprini VTE risk assessment model (RAM) is an individualized risk screening tool that has been implemented in multiple specialties, including general, vascular, plastic surgery, and etc.^14^, ^15^, ^16^, ^17^, ^18^ However, in clinical practice, this classic Caprini risk assessment standard is not suitable for patients with cancer, because according to this standard, the majority patients with cancer in hospital have reached the high risk. The American College of Chest Physicians (ACCP) established a modified Caprini model for VTE risk stratification among patients with abdominal and pelvic cancer to identify candidates that may benefit from prolonged prophylaxis, although no guidelines have been developed for risk‐based extended courses of chemoprophylaxis in thoracic surgery patients.^19^ A modified caprini risk assessment form is recommended for dynamic assessment. Thus, we were prompted to investigate whether the Caprini RAM may be useful in predicting the VTE risk in thoracic surgical patients undergoing thymectomy for thymus or thymic tumor resection.

Therefore, we conducted a single‐center, prospective, observational cohort study that aimed to detect perioperative VTE events that occurred before discharge in patients undergoing thymus or thymic tumor resections at our center who were not treated with VTE prophylaxis and identify those patients who were at high risk of developing VTE.

## Methods

## Patients included in the study

This was a prospective study of all patients who underwent thymectomy at our institution between January 2019 and March 2020. The Institutional Review Board at Beijing Tongren Hospital approved this study, and all patients consented to have their medical records reviewed. The follow‐up period ended upon patient discharge from the hospital.

The definition of a VTE event was any PE or DVT identified by computed tomography pulmonary angiography (CTPA) or duplex proximal lower limb venous ultrasonography. Inclusion criteria comprised: (i) a diagnosis of thymic neoplasms or myasthenia gravis (MG) requiring removal of the thymus; (ii) thymus or thymic tumor resection; (iii) aged 18 years or older; and (iv) documented postoperative follow‐up of 30 days or more. Patients were excluded based on the presence of any of the following criteria: (i) a lack of VTE‐related imaging after surgery or before discharge from the hospital; (ii) more than one postoperative surgical intervention; (iii) the receipt of any perioperative prophylaxis; (iv) confirmed VTE before surgery; and (v) lost to follow‐up. The surgical procedures performed included thoracoscopic surgery and median sternotomy. All patients were mobilized on the first postoperative day or when they left the ventilator. Details of the patients' medical history, including general information (age, sex, body mass index [BMI], hospitalization time and diagnosis); comorbidities (hypertension, diabetes mellitus and coronary atherosclerotic heart disease); and information about the surgical procedure (surgical approach, length of operation and volume of blood loss) were also recorded.

All patients were scored by the modified Caprini RAM used by the Boston Medical Center, and patients were stratified into one of three risk categories: low (scores 0–4), moderate (5–8), or high (nine or more points)^.^ ^16^, ^20^ Lower limb doppler ultrasonography was performed 30 days before surgery and within 30 days after surgery before discharge. Patients with a new diagnosis of postoperative DVT, typical symptoms of PE (chest pain, haemoptysis, dyspnoea or persistent hypoxaemia), or a high Caprini score (≥9) underwent further CTPA examination for PE.

All data were collected prospectively and analyzed using the SPSS statistical software program version 24. Continuous variables were expressed as means and standard deviations. Student's *t*‐test was used to compare the means of the continuous variables between the two groups. Categorical variables were analyzed by or the Mann‐Whitney U test, χ2 tests or Fisher's exact tests (the minimum expected cell size was 5). A *P*‐value ≤ 0.05 indicated statistical significance.

## Results

From the Department of Thoracic Surgery, Beijing Tongren Hospital, Capital Medical University, a total of 237 patients were enrolled in this study. Among these patients, a total of 192 patients, including 103 male patients and 89 female patients, met all the inclusion criteria (Table 1). A total of 45 patients were excluded; 22 had missing records, four received perioperative prophylaxis, two had a complex postoperative course with multiple major operations, and 17 received therapeutic anticoagulation for clinical indications. There were no cases of death. The mean age was 51 years, and postoperative discharge days ranged from three to 35 days.

**Table 1:** Patient characteristics

Variables	Number (%) or mean ± SD
Age(years)	51.13 ± 12.75
Gender	
Male	103 (53.6)
Female	89 (46.4)
Disease type	
Benign	109 (56.8)
Malignancy	83 (43.2)
Comorbiditities	
Hypertension	48 (25.0)
CHD	18 (9.4)
Diabetes	19 (9.9)
BMI >25 (kg/m^2^)	93 (48.4)
Surgical approach	
Minimally invasive surgery	148 (77.1)
Open surgery	44 (22.9)
Length of operation (minutes)	173.18 ± 69.88
Volume of blood loss (mL)	221.04
Myasthenia gravis	127 (66.1)
Central venous catheter	33 (17.2)
Lung disease^†^	59 (30.7)
Confined to bed (>72 hours)	40 (20.8)

BMI, body mass index; CHD, congenital heart disease; SD, standard deviation.

^†^ Abnormal pulmonary function (COPD), pneumonia.

Among the included patients, there were 109 with benign diagnosis and 83 with malignancies (Table 2). Thymic malignancies included 64 (77.1%) thymomas, 13 (15.7%) squamous cell carcinomas, one (1.2%) carcinoid, one (1.2%) lymphoepitheliomatous carcinoma, two lymphoma (2.4.6%) and one (1.2%) asexual cell tumor. The surgical procedures included 44 (39 with malignant diagnosis) median sternotomies and 148 minimally invasive approaches. Among them, 11 cases combined with lung wedge resection, five with resection of pericardium, three with both lung wedge resection and pericardium resection. Mediastinal fat was removed when the patients were diagnosed with MG. There were 127 patients with MG, and 45 combined with thymoma.

**Table 2:** Diagnosis of thymic diseases

	Total (*n* = 192)	VTE (*n* = 17)
Malignancy		
Thymoma	64	9
Thymic carcinoma	13	3
Thymic carcinoid	1	0
Lymphoepitheliomatous carcinoma	1	0
Lymphoma	2	0
Asexual cell tumor	1	0
Benign		
Teratoma	2	0
Thymic hyperplasia	82	4
Thymic cyst	23	1
Lipoma	3	0

VTE, venous thromboembolism.

A total of 17 patients were diagnosed with VTE (Table 3), resulting in an overall incidence of VTE after thymectomy of 8.9% (17 of 192). Among the 17 VTE patients, all were diagnosed with DVT, and none were diagnosed PE. None of the patients had obvious symptoms of VTE (dyspnea or swollen legs). Among the 17 VTE cases, 76.5% (13/17) of the VTE cases were diagnosed within five days after surgery. The median time of postoperative VTE diagnosed was four postoperative days (ranged from two to 15 days).

**Table 3:** Characteristics between the non‐VTE and VTE groups

Variables	Non‐VTE (*n* = 175)	VTE (*n* = 17)	*P*‐value
Age (years)	50.38 ± 12.93	58.82 ± 7.38	0.017
Gender			0.338
Male	92 (52.6)	11 (64.7)	
Female	83 (47.4)	6 (35.3)	
Disease type			0.017
Benign	104 (59.4)	5 (29.4)	
Malignancy	71 (40.6)	12 (70.6)	
Comorbiditities			
Hypertension	43 (24.6)	5 (29.4)	0.883
CHD	14 (8.0)	4 (23.5)	0.097
Diabetes	18 (10.3)	1 (5.9)	0.877
BMI >25 (kg/m^2^)	87 (49.7)	6 (35.3)	0.256
Surgical approach			0.001
Minimally invasive surgery	141 (80.6)	7 (41.2)	
Open surgery	34 (19.4)	10 (58.8)	
Length of operation (minutes)	168.38 ± 66.53	252 ± 119.64	0.002
Volume of blood loss (mL)	173.98 ± 246.33	201.67 ± 302.59	0.304
Myasthenia gravis			0.504
Yes	117 (66.9)	10 (58.8)	
No	58 (33.1)	7 (41.2)	
Central venous catheter	27 (15.4)	6 (35.3)	0.083
Lung disease	52 (29.7)	7 (41.2)	0.328
Confined to bed (>72 hours)	30 (17.1)	10 (58.8)	0.000

BMI, body mass index; CHD, congenital heart disease; VTE, venous thromboembolism.

A total of 12 events (14.5%) occurred in the malignancy group, and five (4.6%) occurred in the benign group (*P* = 0.017). Most of the surgeries were performed by thoracoscopy (77.1%). Specifically, the incidence of VTE in patients who underwent median sternotomy surgery was 22.7% (10 of 44), while the incidence of VTE after minimally invasive surgery was 4.7% (seven of 148).

**Figure 1:**
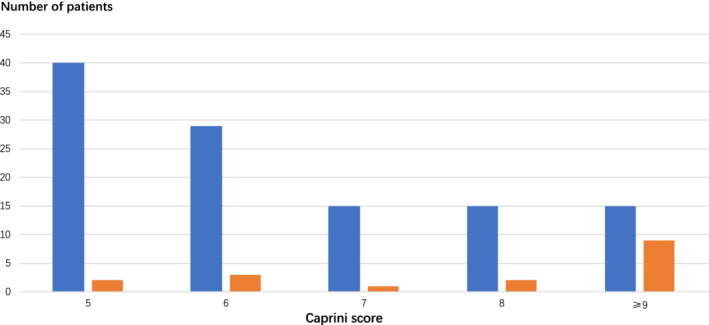
VTE incidence based on Caprini scores. Patients who scored between 5 and 8 points based on the Caprini risk scoring system had a VTE incidence of 7.5%. With scores lower than 5 points, no VTE was observed, however with scores higher than 8 points, an increased incidence of 37.5% was observed. VTE: venous thromboembolism. (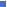) Non‐VTE patients, (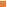) VTE patients

Among the total 192 patients, 61 patients were found to be at low risk (0–4 points), 107 were at moderate risk (5–8 points) and 24 were at high risk (nine points or more). Scores in the low, moderate and high risk groups were associated with VTE incidence rates of 0%, 7.5% (8 of 107) and 37.5% (nine of 24), respectively (*P* < 0.05). In patients diagnosed with thymic malignancy, 10 patients were at low risk, 40 patients were at moderate risk (5–8 points) and 13 patients were at high risk (nine points or more). The corresponding VTE incidence rates for these patients were 0% (0 out of 4), 8.8% (5 of 57) and 31.8% (7 of 22) (*P* < 0.05), respectively (Fig 1). There was obvious incidence discrepancy as the Caprini score increased. VTE did not develop in patients with scores lower than five points. On the contrary, in patients with scores more than eight points, the incidence of VTE was found to be 37.5%, which was consistent with the moderate to high risk group based on the ACCP guidelines.

## Discussion

VTE, comprising DVT and PE, is a well recognized cause of significant perioperative morbidity and mortality. The most recent ACCP and the American Society of Clinical Oncology (ASCO) guidelines on VTE prevention outline perioperative thromboprophylaxis regimens for a broad spectrum of surgical populations, but reference to thoracic surgery patients is noticeably absent.^19^, ^21^ In recent years, studies have shown that the incidence rate of thrombosis after thoracic surgery is high^8^, ^12^, ^13^; however, research has mainly focused on the esophagus and lungs; there are few studies on mediastinal surgery. Mediastinal surgery is very common in thoracic surgery, and thymus‐related surgery is very common in mediastinal surgery. Thus, we are curious about the incidence rate of thrombosis after thymectomy.

Although thymectomy is a kind of thoracic surgery, it is quite different from esophagectomy or lung resection. First, the thymus is an immune organ and most of the patients had systemic immune diseases with or without thymoma, especially myasthenia gravis. Second, thymic malignancy, such as thymoma or thymic carcinoma, is associated with a low degree of malignancy and a comparatively good prognosis. Third, the vast majority of thoracotomy operations are performed by splitting the sternum, which is rarely used in pneumonectomy and esophagectomy. Therefore, we conducted this single‐center prospective cohort study. To the best of our knowledge, this is the first exclusive study of its kind to focus on thoracic surgery.

In this study, all patients were screened for DVT using noninvasive duplex lower‐extremity ultrasonography after surgery, and CTPA was carried out only if patients had typical symptoms of PE, a high Caprini score (≥9 points) or newly diagnosed postoperative DVT. We found that the overall incidence of VTE after thymectomy was 8.9%, which was significantly higher than that reported in previous studies.^8^, ^12^ On further analysis, the incidence of VTE after surgery for thymic malignancy was found to be 14.5%, which was more than twice as high as the incidence of VTE after surgery for benign thymic diseases (4.8%). Because most thymic malignant tumors are addressed with open surgery, the incidence rate of malignant tumors addressed with minimally invasive surgery is only 4.8% (2/42), which is as high as the incidence of VTE after surgery for benign thymic diseases. In addition, we found that the incidence rate of thrombosis after open surgery was 22.7%, which was almost five times that of minimally invasive surgery. This conclusion is different from the previously published results in which median sternotomy was a key risk factor for VTE.

The Caprini RAM has been widely applied for screening patients who are at a high risk of VTE after surgery, and this RAM was also used in this study. Our results showed that the incidence of VTE in the low, moderate and high risk VTE groups were 0%, 7.5% and 37.5%, respectively, which clearly highlights the predictive effectiveness of the Caprini RAM. On further analysis, among patients who underwent thymic malignancy resection, the incidence of VTE in the moderate risk group was found to be 8.8%, while that in the high risk group reached 31.8%, suggesting that additional attention should be given to thymic malignancy patients who are at a high risk of VTE. Additionally, we also noticed that advanced age, a prolonged duration of operation, confinement to a bed for more than 72 hours and central venous catheterization during operation were associated with the occurrence of VTE.

Our study has several strengths. An advantage of this study is that the data was prospectively collected and included specifics on surgical variables. We were able to control for these variables in our analysis, which makes the findings more meaningful. In addition, none of the patients in this study received prophylactic treatment. As such, the results of this study are a good contemporary reflection of the natural situation of the true incidence of VTE. The drawbacks of our study are that the VTE analysis was retrospective and other confounders not previously considered may have impacted the data.

There were several limitations to our study. First, there was no prolonged follow‐up of patients after discharge. Some of the patients may have experienced thrombosis after discharge. In addition, the incidence rate of thymic malignant tumors was low, accounting for only 1% of lung cancer cases; therefore, insufficient data may bias the results.

In conclusion, our study suggests that the incidence of VTE after thymus or thymic tumor surgery is 8.9%. Sternotomy is an important risk factor for VTE after thymectomy, and the Caprini RAM can be used to identify high risk VTE patients.

## Disclosure

The authors declare that there are no conflicts of interest in this study.
